# Very long-term survival in pancreatic cancer

**DOI:** 10.18632/aging.100771

**Published:** 2015-06-25

**Authors:** Marco Dal Molin, Laura D Wood

**Affiliations:** Gastrointestinal & Liver Pathology Department, Johns Hopkins University, Baltimore, MD, USA

Pancreatic ductal adenocarcinoma (PDAC) is often perceived as an incurable disease by both patients and their physicians. While the outcome of most solid tumors has improved over the last 20 years, the median survival for patients with PDAC has steadily remained less than 12 months, even with the best therapeutic regimens currently available [1]. Despite the discouraging statistics, approximately 20% of all patients who undergo surgical resection for PDAC survive five years. Among these, a small percentage experience very long-term survival of more than ten years, and in some cases may even experience complete cure [2, 3].

Interestingly, studies have also indicated that this very long-term survival might not simply be the result of early detection. While patients who survive at least ten years tend to be younger and have more favorable pathological characteristics than “conventional” survivors, very long-term survivors can still present with nodal metastases, poor tumor differentiation, and positive resection margins in up to 40% of cases. This suggests that the atypical clinical behavior of PDAC in these patients may be due to unique tumor biology, rather than clinical features such as earlier stage or more complete surgical resection.

In recent years, numerous DNA sequencing studies have unraveled the complexity of cancer genomes and have provided a molecular rationale for the remarkable heterogeneity that is commonly observed among cancers that share identical pathological features. This concept is exemplified by the recent report of a patient with metastatic bladder cancer who experienced a dramatic response to everolimus, resulting in prolonged survival [4]. Genomic analysis of the patient's cancer identified inactivating mutations in the *TSC1* and *NF2* genes, both of which have been associated with the mTORC1 pathway in preclinical models, thus providing a genetic explanation for the exceptional response and improved survival.

In light of these observations, our group recently analyzed the exomes of a series of well-characterized, pathologically confirmed PDACs from patients who had survived at least ten years after surgical resection of their cancer [5]. Eight carcinomas were subjected to whole-exome sequencing analysis to assess whether somatic mutations in specific genes might explain the atypical clinical course of this particular subgroup of patients with PDAC.

Although a total of 301 somatic mutations were identified in 274 genes (mean of 37.6 non-synonymous somatic mutations per carcinoma), only 5 genes (KRAS, TP53, SMAD4, CDKN2A, and RNF43) were altered in more than one of the eight PDACs. Of these KRAS, TP53, SMAD4, and CDKN2A are previously described commonly mutated driver genes in this tumor type. Interestingly, mutations in RNF43, a tumor suppressor gene that encodes a protein with intrinsic U3 ubiquitin ligase activity, were identified in three of the eight carcinomas (37.5%). Inactivating mutations in this gene have been reported in intraductal papillary mucinous neoplasm (IPMN), a non-invasive pancreatic cystic neo-plasm which has the potential to progress to PDAC [6]. In order to determine whether differences in driver gene mutation frequency underlie very long-term survival, we then analyzed 27 additional PDACs obtained from an independent cohort of very long-term survivors using deep targeted sequencing. To ascertain whether cancers in very long-term survivors arose from cystic neoplasms (as suggested by the *RNF43* mutations), this targeted analysis focused on genes commonly altered in PDAC, IPMN, and other pancreatic cystic neoplasms, including *BRAF, CDKN2A, GNAS, KRAS, PIK3CA, RNF43, SMAD4, TP53,* and *VHL*.

After combining the results of the whole-exome and targeted sequencing analyses, *KRAS* was confirmed to be the most commonly altered gene in PDACs from very long-term survivors (94%), followed by *TP53* (69%), *SMAD4* (26%), and *CDKN2A* (17%). Interestingly, the mutation frequencies of the main PDAC driver genes observed in our cohort was comparable to the frequency that would be expected in conventional PDAC.

Overall, *RNF43* mutations were identified in 11% of the carcinomas, as only 1 of the 27 validation cases harbored a mutation in the gene. Although *RNF43* mutations were relatively frequent in our cohort, a similar frequency has recently been reported by the International Cancer Genome Consortium in non-selected PDACs [7]. The observation that this gene is in fact not mutated at a higher frequency in cancers from very long-term survivors argues against a direct role for *RNF43* in determining improved survival.

Taken together, the results of our study suggest that very long-term survival in pancreatic cancer is unlikely to depend upon small coding somatic mutations in common cancer genes. Although non-genetic mechanisms, such as epigenetic alterations, immunologic differences, or features of the tumor microenvironment, may also be responsible for very long-term survival in patients with PDAC, a genetic basis cannot be excluded altogether. Future studies incorporating analysis of large-scale genomic alterations such as rearrangements, chromothripsis, large deletions or insertions, will be important. Furthermore, it is possible that multiple genes mutated at a low frequency play a role in defining PDACs with less aggressive clinical behavior, perhaps by rendering these cancers more sensitive to therapies or by limiting their metastatic potential. Additional sequencing studies performed on large cohorts of patients, in the context of multi-institutional efforts, will be instrumental in identifying such infrequent genetic events.
